# PP74 Challenges And Potential Improvements In Policy Strategies For Prevention And Early Diagnosis Of Childhood Melanoma: A Patient Perspective

**DOI:** 10.1017/S0266462325102924

**Published:** 2025-12-29

**Authors:** Sonia Muñoz-López, Paula Closa, Pietro Refolo, Costanza Raimondi, Violeta Astratinei, Bettina Ryll, Joan Prades, Dario Sacchini, Josep Maria Borràs, Laura Sampietro-Colom

## Abstract

**Introduction:**

To design healthcare system strategies to inform policy decisions in prevention and early diagnosis of melanoma in children, adolescents, and young adults (CAYA) in Europe, a patient perspective is needed. The MELCAYA project addresses this issue, since this disease lacks preventive and diagnostic strategies. A focus session was conducted to identify challenges and potential improvements in current policies on rare cancers.

**Methods:**

The focus session involved 15 participants, including patients, caregivers, patient advocates, and healthcare professionals from seven European countries. This session explored, through open discussions, challenges in melanoma prevention and early diagnosis as well as the role of patient advocates in policy development and implementation. Furthermore, a group dynamic was carried out to collect patients’ insights on 20 policy strategies from National Cancer Control Plans (NCCP), which were categorized into three areas: (i) early diagnosis and symptom awareness; (ii) health promotion and ultraviolet radiation prevention; and (iii) precision medicine and genetic counseling. Participants provided insights by identifying lacking policies and proposing actions for their implementation.

**Results:**

Key challenges in melanoma prevention and early diagnosis include: (i) low awareness and education among the general population and a misconception about its severity; (ii) fragmented care in the patient journey from diagnosis to treatment; (iii) ineffective communication with younger generations; and (iv) difficulties on how to navigate through the healthcare systems. Participants proposed 18 additional potential strategies to the NCCP and 22 actions for policy integration into healthcare systems. The current ad hoc approach fails to fully leverage contributions from advocates and healthcare professionals, often resulting in policies misaligned with clinical realities and patient needs.

**Conclusions:**

This focus session provided patient ideas for policy initiatives, identified gaps in current policies from the NCPP, and emphasized the need for improved awareness, education, and communication strategies. Patient involvement in policy development, in a more structured way, is crucial for creating effective strategies, and its integration into healthcare systems could enhance melanoma prevention and early diagnosis in CAYA populations.

